# Knowledge, Perceived Barriers, and Whole‐Grain Bread Consumption among University Students in Southwest Iran in the 2024–2025 Academic Year: A Cross‐Sectional Study

**DOI:** 10.1002/hsr2.72660

**Published:** 2026-06-16

**Authors:** Ali Keshavarz, Fatemeh Borazjani, Faezeh Moeini Badi, Kambiz Ahmadi Angali, Mohammad Parsa Bayat, Samaneh Hajjarzadeh

**Affiliations:** ^1^ Student Research Committee Ahvaz Jundishapur University of Medical Sciences Ahvaz Iran; ^2^ Nutrition and Metabolic Diseases Research Center, Clinical Sciences Research Institute Ahvaz Jundishapur University of Medical Sciences Ahvaz Iran; ^3^ Department of Nutrition, School of Allied Medical Sciences Ahvaz Jundishapur University of Medical Sciences Ahvaz Iran; ^4^ Social Determinants of Health Research Center Ahvaz Jundishapur University of Medical Sciences Ahvaz Iran; ^5^ Department of Biostatistics and Epidemiology, School of Health Science Ahvaz Jundishapur University of Medical Sciences Ahvaz Iran; ^6^ Diabetes Research Center, Health Research Institute Ahvaz Jundishapur University of Medical Sciences Ahvaz Iran

**Keywords:** bread, cross‐sectional, dietary habit, knowledge, student

## Abstract

**Background and Aims:**

Whole‐grain bread is rich in fiber and micronutrients, yet student consumption is low and linked to poor health outcomes. This study evaluates students' nutritional knowledge, perceived barriers to whole‐grain bread consumption, and overall dietary habits at Ahvaz Jundishapur University of Medical Sciences.

**Methods:**

A cross‑sectional study of 567 students (408 undergraduates, 159 postgraduates) assessed knowledge, barriers, and dietary habits using a validated questionnaire. Two trained nutritionists conducted face‑to‑face interviews. The questionnaire was pretested in 50 students; the barriers section showed high reliability (α = 0.88) and CVI/CVR = 1.

**Results:**

Of 567 students, 239 (42.15%) rarely consumed whole‑grain bread, 144 (25.4%) consumed it 1–2 times per month, and 184 (32.45%) consumed it ≥ 1–3 times per week. Mean knowledge and barrier scores were 13.88 and 18.53, respectively. Significant associations were found between whole‑grain consumption and education status (*p* = 0.04), knowledge (< 0.001), barriers (*p* < 0.001), sugar‑sweetened beverage (SSB) intake (*p* = 0.04), and the number of main meals per day (*p* = 0.006). Positive correlations with knowledge (*r* = 0.196) and negative correlations with barriers (*r* = −0.422) were observed (both *p* < 0.001). Higher knowledge was associated with greater dairy intake (OR = 1.194; 95% CI: 1.079–1.321), while barriers correlated negatively with vegetable (OR = 0.943; 95% CI: 0.907–0.980), fruit (OR = 0.941; 95% CI: 0.910–0.974), and dairy intake (OR = 0.938; 95% CI: 0.906–0.972), and positively with sweet/salty snacks (OR = 1.067; 95% CI: 1.029–1.105).

**Conclusion:**

Students showed moderate to high awareness of whole‐grain bread benefits; however, low consumption was associated with accessibility and price. Higher whole‐grain bread intake was associated with better overall dietary quality.

## 1 Introduction

Bread is a staple food widely consumed throughout the day and serves as a major source of energy in many populations worldwide [1]. The market today offers a wide range of bread options, including whole‐grain and white varieties, to accommodate different preferences and nutritional needs [2]. Whole‐grain bread is considered nutritionally superior due to its higher content of dietary fiber, B vitamins, iron, magnesium, zinc, and other essential micronutrients [3]. These nutrients contribute to the prevention of several chronic conditions [4] including hypertension [5], overweight and obesity [6], cardiovascular disease [7], certain types of cancer [8], and type 2 diabetes [9]. Despite these well‐established benefits, whole‐grain bread consumption among university students is quite low, similar to that of the general population [10]. This highlights the need for initiatives to boost whole grain consumption to meet the Dietary Guidelines for Americans' recommendation of 50% of total grain intake [11]. A study in the United States reported that students consume only about 12% of the recommended minimum amount of whole‑grain bread [12]. Similar findings have been reported in Iran, where 29.7% of students at Fasa University do not consume whole‑grain bread at all and instead prefer refined bread products [13]. This pattern is concerning, as the university years are one of the most critical periods during which long‐term dietary habits are formed [14].

Multiple factors, including environmental, cultural, social, economic, and institutional aspects (e.g., promotion of industrial foods and limited whole‐grain availability in cafeterias), along with barriers such as inadequate nutritional awareness, financial constraints, lifestyle changes, and increased reliance on processed foods, influence dietary patterns and reduce whole grain intake among university students [13, 14, 15, 16]. A reduced intake of whole grain is potentially associated with adverse health outcomes, including elevated cholesterol, weight gain, and diminished cognitive performance [17, 18, 19]. Consequently, dietary habits established during university may have long‐term individual and public health implications [20].

Improving knowledge programs and university‐level policy changes can increase whole‐grain bread access and consumption [13].

Earlier studies applying frameworks such as the Theory of Planned Behavior have typically examined knowledge, barriers, or behavior toward whole‐grain products in isolation [15, 20, 21]. While earlier studies often examined knowledge, barriers, or behavior separately, this study jointly analyzes these factors with actual whole‑grain bread consumption and broader dietary habits among university students to clarify their collective association with intake and diet quality.

## 2 Methods

### 2.1 Study Design and Participants

The participants of the current cross‐sectional study consisted of undergraduate and postgraduate students (including both Master's and Ph.D. candidates) from different faculties of Ahvaz Jundishapur University of Medical Sciences, located in southwestern Iran.

The entire study was conducted between November 2024 and March 2025. The participants were recruited using a convenience sampling method considering time limitations, logistical feasibility, and accessibility of the target population within the university setting. Eligibility criteria were: 1) enrollment as a student at any faculty of Ahvaz Jundishapur University of Medical Sciences; 2) age ≥ 18 years; and 3) willingness to participate in the study and provide written informed consent. Exclusion criteria were: 1) adherence to a self‐reported ketogenic diet or a strict vegan diet, as these dietary patterns fundamentally alter the type and quantity of bread consumed independent of general attitudes or knowledge; 2) self‐reported Celiac disease or gluten sensitivity (no cases identified); and 3) incomplete or inconsistent questionnaire responses. First, the eligible students were informed of the study goals and procedure. Second, they were asked to read and signed a written consent form and were informed that data confidentiality was assured.

The study followed the Declaration of Helsinki and was approved by the Ethics Committee of Ahvaz Jundishapur University of Medical Sciences (IR.AJUMS.REC.1404.137).

### 2.2 Sample Size

The required sample size was calculated using the below single population proportion formula for descriptive cross‐sectional studies [22].

$$n=\frac{Z^{2}\times P\;\left(1-P\right)}{d^{2}}$$

Considering a 95% confidence level (Z = 1.96), an expected prevalence of 50% (*p* = 0.5) to account for maximum variability, and a margin of error of 5% (d = 0.05), the initial sample size was estimated at 384 students.

$$n=\frac{1.96^{2}\times 0.5\;\left(1-0.5\right)}{0.05^{2}}=384.16$$

To compensate for potential non‐response or incomplete questionnaires, a 20% increase was applied, resulting in a final minimum required sample size of 461 participants. In practice, due to the high level of cooperation from students and to further increase the statistical power and generalizability of the findings, a total of 567 participants (408 undergraduate and 159 postgraduate students) were recruited during the study procedures.

### 2.3 Data Collection and Questionnaire Content

Data were collected using a set of structured questionnaires, which were administered through face‐to‐face interviews by two trained nutritionists (A.K. and M.P.B.). Before the main survey, the nutritionists received a dedicated training session to ensure standardization of interview procedures and uniformity in data collection.

### 2.4 Demographic and Anthropometric Questionnaire

The **Demographic and Anthropometric Questionnaire** was designed to capture information on age, sex, educational level (undergraduate or postgraduate), self‐reported weight and height (for BMI calculation), duration of daytime and nighttime sleep, and history of non‐communicable diseases.

### 2.5 Knowledge, Barriers, and Whole‐Grain Bread Consumption Questionnaire

A comprehensive literature review was conducted to develop the initial version of the "**Knowledge, barriers, and qualitative** whole‐grain bread **consumption questionnaire**". The draft version was reviewed by two expert nutritionists and two **health education specialists** to assess face and content validity. Based on their feedback, the final questionnaire was structured as follows: one Likert‐scale question assessing qualitative whole‐grain bread consumption (rarely/never, 1–2 times per month, 1–3 times/week, and ≥ 3 times per week). Due to the limited number of students reporting whole‐grain bread consumption in the two highest frequency categories (1–2 times per week and ≥ 3 times per week), these groups were merged into a single category (≥ 1–3 times per week) for subsequent analyzes. one item on the source of whole‑grain bread accessibility (home‐baked bread, traditional bakery bread, unlabeled traditional packaged bread (e.g., Tiri, Lavash) or industrial bakery bread), seven Likert‐scale questions assessing potential barriers (taste, color, affordability, texture, gastrointestinal complications, and accessibility) and four Likert‐scale questions assessing knowledge and perception. Questions 1 and 2 in the knowledge section were dedicated to assessing the participants' ability to distinguish whole‑grain bread from white bread, while questions 3 and 4 evaluated their knowledge regarding the health benefits of whole‑grain bread consumption. Responses to knowledge and barrier questions were rated on a five‐point Likert scale (ranging from “strongly agree” to “strongly disagree”).

The final questionnaire (Table 1) was pretested in a pilot study of 50 students to evaluate clarity, feasibility, and reliability. The Perceived Barriers construct demonstrated excellent internal consistency (Cronbach's α = 0.88), exceeding the conventional threshold of 0.70 for exploratory research [23]. For the Knowledge construct, the Cronbach's α coefficient was 0.54.

**Table 1:** Items of knowledge, barriers, and qualitative whole‐grain bread consumption questionnaire.

Section	Questions	Response options with its' scores
Quantitative whole‐grain bread consumption & Source of accessibility	How often do you consume whole‐grain bread?	Rarely use, 1–2 times per month, 1–2 times per week, ≥ 3 times per week
	Where do you usually obtain your whole‐grain bread?	home‐baked bread, traditional bakery bread, unlabeled traditional packaged bread (e.g., Tiri, Lavash), industrial bakery bread
Knowledge and perception (Likert 5‐point)	1. Any dark‐colored bread is whole grain	Strongly agree (1), Agree (2), Neutral (3), Disagree (4), Strongly disagree (5)
	2. Only bread made from unrefined wheat flour is whole‐bread	Strongly agree (5), Agree (4), Neutral (3), Disagree (2), Strongly disagree (1)
	3. From a health perspective, it makes no difference whether we eat whole‐grain bread or white‐bread	Strongly agree (1), Agree (2), Neutral (3), Disagree (4), Strongly disagree (5)
	4. People who consume whole‐grain bread are less likely to develop overweight and chronic diseases (heart disease, diabetes, hypertension, etc.)	Strongly agree (5), Agree (4), Neutral (3), Disagree (2), Strongly disagree (1)
Barrier (Likert 5‐point)	5. I do not prefer whole‐grain bread because of its taste.	Strongly agree (5), Agree (4), Neutral (3), Disagree (2), Strongly disagree (1)
	6. I do not prefer whole‐grain bread because of its dark color.	Strongly agree (5), Agree (4), Neutral (3), Disagree (2), Strongly disagree (1)
	7. I do not prefer whole‐grain bread because it is more expensive.	Strongly agree (5), Agree (4), Neutral (3), Disagree (2), Strongly disagree (1)
	8. I do not prefer whole‐grain bread because it is hard or of low quality	Strongly agree (5), Agree (4), Neutral (3), Disagree (2), Strongly disagree (1)
	9. I do not prefer whole‐grain bread because it has a short shelf‐life	Strongly agree (5), Agree (4), Neutral (3), Disagree (2), Strongly disagree (1)
	10. I do not prefer whole‐grain bread because it causes bloating or gastrointestinal side effects	Strongly agree (5), Agree (4), Neutral (3), Disagree (2), Strongly disagree (1)
	11. Whole‐grain bread bakeries or sales centers are not sufficiently accessible to me	Strongly agree (5), Agree (4), Neutral (3), Disagree (2), Strongly disagree (1)

While α = 0.54 is below the 0.70 threshold for reflective scales, this is expected for a formative index where items collectively define the construct rather than reflect a single latent trait. The Knowledge items assessed two distinct domains: visual identification (Q1–2) and health benefit awareness (Q3–4). In formative models, high inter‐item correlation is not required, rendering Cronbach's α an inappropriate reliability metric [24]. Accordingly, the four knowledge items were treated as a formative index capturing distinct dimensions of whole‑grain bread awareness (visual identification and health benefit knowledge). The total knowledge score was therefore computed as the unweighted sum of the four items, representing overall knowledge breadth rather than a unidimensional latent trait.

The content validity of the questionnaire was rigorously established, with both content validity index (CVI) and content validity ratio (CVR) equal to 1.0, confirming that the items adequately represent the content domain of whole‐grain bread knowledge and barriers [25].

Knowledge and barrier scores were calculated as the sum of Likert‐scale responses for the respective items, as shown in Table 1.

### 2.6 Dietary Habits Questionnaire

In addition, a **dietary habits questionnaire** comprising 17 Likert‐type questions was used to assess the frequency of consumption of major food groups (vegetables, fruits, bread, rice, pasta, white meat, red meat, eggs, dairy products, pulses, and sweet/salty snacks) as well as dietary habits such as type of oils commonly used in food preparation, typical beverages consumed with main meals, number of meals and snacks per day, and breakfast consumption. Intake frequencies were categorized into four levels: every day, ≥ 4 times per week, 1–3 times per week, and rarely use.

### 2.7 Statistical Analysis

Statistical analyzes were performed using IBM SPSS Statistics for Windows, Version 22.0 (IBM Corp., Armonk, NY, USA) [26]. The Kolmogorov‐Smirnov test and visual inspection of histograms were used to assess the normality of continuous variables [27]. Normally distributed continuous variables are presented as mean ± standard deviation (SD), while categorical variables are presented as frequencies and percentages [28]. For comparisons between qualitative categories of whole‐grain bread consumption, one‐way analysis of variance (ANOVA) was used for continuous variables, and the Chi‐square test was used for categorical variables [29]. Pearson or Spearman correlation coefficients were calculated to assess associations between whole‐grain bread consumption and other variables, as appropriate [30]. Associations between knowledge/barrier scores and food group intake were evaluated using ordinal logistic regression with a cumulative logit link, as each food group was measured on a four‐level ordinal scale (every day, ≥ 4 times/week, 1–3 times/week, rarely). The reported odds ratios (ORs) are cumulative ORs, representing the odds of being in a higher intake category per one‐unit increase in the predictor. Model 1 was unadjusted and Model 2 was adjusted for age, BMI, weight, sex, and education level. All statistical tests were two‐sided, and a *p*‐value < 0.05 was considered statistically significant [28]. All primary analyzes (group comparisons, correlations, and regression models) were prespecified. The cross‑tabulation quantifying the knowledge–action gap (Table 5) was not a prespecified hypothesis test and is presented as a descriptive summary.

## 3 Results

### 3.1 Characteristics of the Study Population

The 567 participants (mean age 22.66 ± 4.75 years; BMI 23.85 ± 5.66 kg/m^2^) were predominantly female (*n* = 332, 58.6%) and undergraduate (*n* = 408, 72.0%); 159 (28.0%) pursued postgraduate degrees (MSc and Ph.D.). Only educational status differed significantly across consumption categories (*p* = 0.04) (Table 2) and was subsequently adjusted for in multivariate models (Table 6).

**Table 2:** Demographic, anthropometric, knowledge and barrier score status of students stratified by qualitative whole‐grain bread consumption.

Variables	Total *N* = 567	Qualitative categories of whole‐grain bread consumption			*p*‐value	Correlation with whole‐grain bread consumption (*r*, *p*‐value)
		Rarely use (*N* = 239)	1–2 times per month (*N* = 144)	≥ 1–3 times per week (*N* = 184)		
Age (year) mean(SD)	22.66 (4.75)	22.85 (5.06)	22.47 (3.72)	22.55 (5.05)	0.70*	(−0.048, 0.26)
Sex *n* (%)						
Male	235 (41.4)	102 (43.4)	55 (23.4)	78 (33.2)	0.66^#^	(0.007, 0.88)
Female	332 (58.6)	137 (41.3)	89 (26.8)	106 (31.9)		
Education status *n* (%)						
Undergraduate	408 (72.0)	169 (41.4)	115 (28.2)	124 (30.4)	0.04^#^	(0.021, 0.62)
Post‐graduate	159 (28.0)	70 (57.4)	29 (23.8)	60 (37.7)		
Weight (kg) mean (SD)	68.31 (15.23)	67.36 (14.39)	68.66 (15.81)	69.26 (15.82)	0.42*	(0.046, 0.28)
BMI (kg/m^2^) mean (SD)	23.85 (5.66)	23.69 (5.36)	23.97 (6.0)	23.97 (5.79)	0.85*	(0.008, 0.85)
Day‐time sleep (hr) mean (SD)	1.54 (1.85)	1.71 (1.99)	1.56 (1.86)	1.32 (1.62)	0.11*	(−0.10, 0.02)
Night‐time sleep (hr) mean (SD)	6.72 (1.42)	6.60 (1.41)	6.67 (1.39)	6.91 (1.44)	0.07*	(0.088, 0.04)
Chronic disease history (positive) *n* (%)						
Diabetes or pre‐diabetes	3	2 (66.7)	1 (33.3)	0	0.48^#^	(−0.048, 0.26)
Cardiovascular diseases	8	1 (12.5)	3 (37.5)	4 (50.0)	0.65^#^	(−0.003, 0.95)
Digestive diseases	52	4 (7.7)	13 (25.0)	35 (67.3)	0.69^#^	(0.055, 0.19)
Fatty liver	16	1 (6.3)	6 (37.5)	9 (56.3)	0.42^#^	(0.057, 0.17)
Respiratory disease	5	1 (20.0)	1 (20.0)	3 (60.0)	0.82^#^	(−0.013, 0.75)
Others disease	38	5 (13.2)	8 (21.1)	25 (65.8)	0.86^#^	(−0.062, 0.14)
Knowledge score mean (SD)	13.88 (1.75)	13.52 (1.72)	13.99 (1.67)	14.25 (1.77)	< 0.001*	(0.196, < 0.001)
Barrier score mean (SD)	18.53 (5.14)	20.72 (4.66)	18.68 (4.40)	15.60 (4.84)	< 0.001*	(−0.422, < 0.001)
Typical drinking beverages with meals (used) *n* (%)						
Sugar‐sweetened beverages (SSBs)	91 (16.0)	49 (53.8)	17 (18.7)	25 (27.5)	0.04^#^	(−0.087, 0.04)
Water	258 (45.4)	98[31]	77 (29.8)	83 (32.2)	0.06^#^	(0.045, 0.29)
Soda water	50 (8.8)	25 (50)	14 (28)	11 (22)	0.25^#^	(−0.065, 0.12)
Dough or kefir	66 (11.6)	23 (34.8)	16 (24.2)	27 (40.9)	0.27^#^	(0.066, 0.12)
No drinks	56 (9.9)	26 (46.4)	10 (17.9)	20 (35.7)	0.39^#^	(−0.006, 0.88)
Regular cooking oil (used) *n* (%)						
Solid oil/animal fat/butter	51 (9.0)	23 (45.1)	10 (19.6)	18 (35.3)	0.61^#^	(−0.002, 0.96)
Liquid oil	224 (39.4)	97 (43.3)	56 (25)	71 (31.7)	0.90^#^	(−0.018, 0.67)
Frying oil	144 (25.4)	65 (45.1)	38 (26.4)	41 (28.5)	0.49^#^	(−0.047, 0.27)
Daily meals/snacks number						
Snacks per day	1.92 (1.18)	1.91 (1.34)	1.88 (0.979)	1.96 (1.09)	0.81*	(0.048, 0.26)
Main meals per day	2.73 (0.52)	2.68 (0.53)	2.68 (0.61)	2.83 (0.42)	0.006*	(0.124, 0.003)
Source of whole‐grain bread accessibility (used) *n* (%)						
Home‐baked bread	94 (16.5)	36 (38.3)	18 (19.1)	40 (42.6)	0.06^#^	(0.070, 0.10)
Traditional bakery bread	314 (55.3)	116 (36.9)	91 (29)	107 (34.1)	0.01^#^	(0.092, 0.03)
Unlabeled traditional packaged bread	103 (18.1)	49 (47.6)	27 (26.2)	27 (26.2)	0.30^#^	(−0.063, 0.13)
Industrial bakery bread	145 (25.5)	70 (48.3)	31 (21.4)	44 (30.3)	0.20^#^	(−0.058, 0.17)

*Note:* The quantitative and qualitative variables are reported as mean (SD) and frequency (percent), respectively. *p‐value based on One‐way ANOVA test. ^#^ *p*‐value based on Chi‐square test. Bold values indicate statistically significant results (*p* < 0.05).

### 3.2 Whole‐Grain Bread Consumption Patterns and Dietary Habits

Of the 567 participants, 239 (42.15%) reported rare intake of whole‑grain bread, 144 (25.4%) reported consumption 1–2 times per month, and 184 (32.45%) reported intake ≥ 1–3 times per week. The mean knowledge score was 13.88 ± 1.75 (range: 4–20), and the mean barrier score was 18.53 ± 5.14 (range: 7–35) (Table 2). Additionally, mean knowledge and barrier scores did not differ significantly between males (13.80 ± 1.74 and 18.28 ± 4.99) and females (13.94 ± 1.76 and 18.72 ± 5.25) (*p* = 0.37 and 0.31, respectively) (Table 3).

**Table 3:** Gender‐based comparison of knowledge and perceived barrier scores among university students.

Variable	Males	Females	*p*‐value*
Knowledge score	13.80 ± 1.74	13.94 ± 1.76	0.37
Barrier score	18.28 ± 4.99	18.72 ± 5.25	0.31

*Note:* *Data are presented as mean ± SD. *p*‐value from independent samples *t*‐test.

As shown in Table 4, whole‐grain bread consumption differed significantly across intake categories for vegetables (*p* = 0.008), fruits (*p* < 0.001), dairy (*p* = 0.006), eggs (*p* = 0.02), pulses (*p* < 0.001), and sweet/salty snacks (*p* = 0.02). Positive Spearman correlations were observed for vegetables, fruits, dairy, white and red meat, eggs, and pulses (*r* range = 0.088–0.207, all *p* < 0.05), whereas sweet/salty snacks correlated negatively (*r* = −0.137, *p* = 0.001). No associations were found for pasta or rice.

**Table 4:** Associations between qualitative categories of whole‐grain bread consumption and qualitative food groups intake (Chi‐square test and Spearman correlation).

Food groups	Chi‐square *p*‐value	Spearman correlation (*r*, *p*‐value)
Vegetables	**0.008**	(0.170, **< 0.001)**
Fruits	**< 0.001**	(0.207, **< 0.001)**
Dairy	**0.006**	(0.121, **0.004)**
Rice	0.31	(−0.058, 0.17)
Pasta	0.98	(0.004, 0.92)
White meat	0.14	(0.102, **0.02)**
Red meat	0.14	(0.088, **0.04)**
Egg	**0.02**	(0.119, **0.005)**
Pulses	**< 0.001**	(0.158, **< 0.001)**
Sweet/salty snack	**0.02**	(−0.137, **0.001)**

*Note:* Bold values indicate statistically significant results (*p* < 0.05).

### 3.3 Nutritional Knowledge and Perceived Barriers

The majority of students demonstrated awareness of the health benefits of whole‑grain bread, with 414 of 567 (73.0%) correctly disagreed that there is no difference between whole‑grain and white bread (Q3), and 340 of 567 (60.1%) correctly agreed that whole‑grain bread reduces the risk of overweight and chronic diseases (Q4). However, most participants were unable to correctly distinguish whole‑grain bread from white bread, as about 62% and 63% answered questions 1 and 2 incorrectly, respectively. Notably, correct response to Q3 was significantly associated with higher whole‑grain bread consumption (*p* = 0.001), whereas correct response to Q4 was uniformly high across all consumption groups and showed no significant association (*p* = 0.401) (Table 5). This indicates that while health benefit awareness is widespread, practical identification skills are selectively higher among regular consumers. The most common barriers were limited accessibility (387/567, 68.3%), hard texture or low quality (320/567, 56.4%), higher price (308/567, 54.4%), unfavorable taste (290/567, 51.1%), short shelf‑life (277/567, 48.8%), dark color (273/567, 48.1%), and gastrointestinal side effects (243/567, 42.8%).

**Table 5:** Knowledge of whole‐grain bread health benefits by consumption frequency.

Knowledge item	Response^#^	Total (*N* = 567) *n* (%)	Qualitative categories of whole‐grain bread consumption			*p*‐value*
			Rarely use (*n* = 239) *n* (%)	1–2 times/month (*n* = 144) *n* (%)	≥ 1–3 times/week (*n* = 184) *n* (%)	
Q3. From a health perspective, it makes no difference whether we eat whole‐grain bread or white‐bread.	Agree (incorrect)	42 (7.4)	26 (61.9)	7 (16.7)	9 (21.4)	0.001
	Neutral	111 (19.5)	59 (53.2)	28 (25.2)	24 (21.6)	
	Disagree (correct)	414 (73.0)	154 (37.2)	109 (26.3)	151 (36.5)	
Q4. People who consume whole‐grain bread are less likely to develop overweight and chronic diseases (heart disease, diabetes, hypertension, etc.)	Agree (correct)	340 (60.1)	155 (45.6)	81 (23.8)	104 (30.6)	0.40
	Neutral	183 (32.3)	68 (37.4)	49 (26.9)	65 (35.7)	
	Disagree (incorrect)	43 (7.6)	16 (37.2)	13 (30.2)	14 (32.6)	

*Note:* *p‐value from Chi‐square test for independence across the three consumption groups. # The original 5‑point Likert scale (“strongly agree” to “strongly disagree”) was collapsed into three categories: Agree (strongly agree + agree), Neutral, and Disagree (disagree + strongly disagree), to avoid sparse cells and summarize response patterns. Bold values indicate statistically significant results (*p* < 0.05).

### 3.4 Knowledge‐Action Gap

A marked knowledge–action gap was observed (Table 5). Among the 340 students who correctly acknowledged the health benefits of whole‑grain bread (Q4 correct), 155 (45.6%) consumed it rarely and 81 (23.8%) only 1–2 times per month; thus, over two‑thirds of fully health‑aware students reported infrequent intake. A similar pattern emerged for those who correctly identified that whole‑grain bread differs from white bread (Q3 correct, *n* = 414); of these, 263 (63.5%) were rare or occasional consumers. The non‑significant association between Q4 and consumption frequency (*p* = 0.401) further confirms that awareness of long‑term health advantages alone does not translate into regular consumption, underscoring the decisive role of sensory, economic, and accessibility barriers.

### 3.5 Factors Associated With Whole‐Grain Bread Consumption

As shown in Table 2, education level (*p* = 0.04), knowledge and barrier scores (both *p* < 0.001), sugar‐sweetened beverages (SSB) intake (*p* = 0.04), number of main meals per day (*p* = 0.006), and sourcing from traditional bakeries (*p* = 0.01) differed significantly across consumption categories. Whole‑grain bread consumption correlated positively with knowledge (*r* = 0.196, *p* < 0.001) and negatively with barriers (*r* = −0.422, *p* < 0.001). Beyond these, statistically significant but trivial correlations were observed for daytime sleep (*r* = −0.10, *p* = 0.02), nighttime sleep (*r* = 0.088, *p* = 0.04), number of main meals per day (*r* = 0.124, *p* = 0.003), and traditional bakeries (*r* = 0.092, *p* = 0.03); given the negligible effect sizes, these associations are unlikely to hold practical importance. No statistically significant correlations were found for age, sex, weight, BMI, history of chronic diseases, type of regular cooking oil, number of daily snacks, or other sources of whole‑grain bread accessibility (all *p* > 0.05).

To further clarify the link with broader dietary habits, we examined Knowledge and Barrier Scores in relation to food group intake using ordinal regression (Table 6). In the fully adjusted model, higher Knowledge Scores were significantly associated with a greater likelihood of dairy intake (OR = 1.194; 95% CI: 1.079–1.321; *p* = 0.001). Barrier Scores showed negative associations with vegetable (OR = 0.943; 95% CI: 0.907–0.980; *p* = 0.003), fruit (OR = 0.941; 95% CI: 0.910–0.974; *p* < 0.001), and dairy intake (OR = 0.938; 95% CI: 0.906–0.972; *p* < 0.001), and a positive association with sweet/salty snacks (OR = 1.067; 95% CI: 1.029–1.105; *p* < 0.001). No significant associations were found for rice, pasta, white meat, red meat, eggs, or pulses (*p* > 0.05). Notably, in the unadjusted model, higher barrier scores were associated with lower egg intake (OR = 0.955; 95% CI: 0.920–0.992; *p* = 0.02); however, this association attenuated and became non‑significant after covariate adjustment (OR = 0.966; 95% CI: 0.929–1.005; *p* = 0.09).

**Table 6:** The association of students' knowledge and barrier scores with qualitative food groups intake.

Food groups	OR (95% CI)	SE	*p*‐value^@^
Knowledge score			
Vegetables			
Model 1	1.009 (0.904–1.127)	0.056	0.87
Model 2	1.007 (0.902–1.125)	0.056	0.90
Fruits			
Model 1	1.067 (0.971–1.172)	0.048	0.18
Model 2	1.063 (0.966–1.169)	0.048	0.21
Dairy			
Model 1	1.193 (1.079–1.319)	0.051	0.001
Model 2	1.194 (1.079–1.321)	0.052	0.001
Rice			
Model 1	1.099 (0.954–1.266)	0.072	0.19
Model 2	1.108 (0.961–1.276)	0.072	0.16
Pasta			
Model 1	0.880 (0.633–1.223)	0.168	0.45
Model 2	0.865 (0.615–1.217)	0.174	0.41
White meat			
Model 1	1.074 (0.928–1.242)	0.074	0.34
Model 2	1.077 (0.931–1.247)	0.075	0.32
Red meat			
Model 1	0.947 (0.841–1.067)	0.061	0.37
Model 2	0.940 (0.834–1.060)	0.061	0.31
Egg			
Model 1	1.096 (0.982–1.223)	0.056	0.10
Model 2	1.102 (0.984–1.235)	0.058	0.09
Pulses			
Model 1	0.950 (0.845–1.068)	0.60	0.39
Model 2	0.962 (0.853–1.084)	0.061	0.52
Sweet/salty snacks			
Model 1	0.919 (0.833–1.014)	0.50	0.09
Model 2	0.914 (0.826–1.011)	0.051	0.08
Barrier score			
Food groups			
Vegetables			
Model 1	0.941 (0.906–0.978)	0.020	0.002
Model 2	0.943 (0.907–0.980)	0.020	0.003
Fruit			
Model 1	0.938 (0.908–0.970)	0.017	< 0.001
Model 2	0.941 (0.910–0.974)	0.017	< 0.001
Dairy			
Model 1	0.939 (0.907–0.972)	0.018	< 0.001
Model 2	0.938 (0.906–0.972)	0.018	< 0.001
Rice			
Model 1	1.006 (0.959–1.055)	0.024	0.82
Model 2	1.005 (0.958–1.056)	0.025	0.83
Pasta			
Model 1	1.082 (0.969–1.209)	0.057	0.16
Model 2	1.070 (0.958–1.195)	0.056	0.23
White meat			
Model 1	0.995 (0.947–1.046)	0.025	0.86
Model 2	0.993 (0.945–1.044)	0.026	0.79
Red meat			
Model 1	0.997 (0.958–1.038)	0.021	0.89
Model 2	1.002 (0.962–1.043)	0.021	0.942
Egg			
Model 1	0.955 (0.920–0.992)	0.019	0.02
Model 2	0.966 (0.929–1.005)	0.020	0.09
Pulses			
Model 1	0.999 (0.960–1.040)	0.020	0.97
Model 2	1.007 (0.966–1.049)	0.021	0.76
Sweet/salty snacks			
Model 1	1.068 (1.031–1.106)	0.018	< 0.001
Model 2	1.067 (1.029–1.105)	0.018	< 0.001

*Note:* Model 1: unadjusted; Model 2: adjusted for age, BMI, weight, sex, and education levels. @ *p*‐value based on GLM regression. Bold values indicate statistically significant results (*p* < 0.05).

## 4 Discussion

This study offers novel insights into university students' knowledge, self‐reported consumption frequency, perceived barriers, and dietary correlates of whole‑grain bread intake. Substituting refined bread with whole‑grain bread is widely recognized as an effective strategy for improving diet quality and preventing chronic diseases [32, 33, 34]. In particular, whole‑grain bread consumption has been consistently associated with lower risks of obesity, type 2 diabetes, cardiovascular disease, and certain cancers [35, 36, 37, 38].

### 4.1 Whole‐Grain Bread Consumption and Nutritional Knowledge

Our findings revealed that the frequency of whole‐grain bread consumption among students was generally low. Most participants reported rare intake (≈ 42%) or consumption only 1–2 times per month (≈ 25%), while refined breads were the predominant type consumed. Knowledge scores regarding whole‐grain bread consumption were significantly lower among non‐consumers compared with weekly consumers (Table 2); however, this difference was driven primarily by the ability to distinguish whole‐grain from white bread (Q3, *p* = 0.001), whereas awareness of health benefits (Q4) was uniformly high across all consumption groups (Table 5).

The limited consumption observed in this study aligns with previous reports. Zanini et al. (2023) conducted a cross‐sectional study in northern Italy to examine perceptions of health benefits, barriers, and the frequency of whole grain consumption [31]. They found that participants generally had positive attitudes toward whole grain products, particularly concerning their health benefits. However, only 11% of participants consumed whole grain foods daily, as recommended by the Italian dietary guidelines, while 34% were non‐consumers [31]. Similarly, Ariya et al. (2022) reported that among Iranian students, 53% preferred refined bread over whole‑grain bread [13]. Based on another qualitative study conducted among Cornell University students in the United States, although the majority of participants believed that including whole‐grain pasta in their diet was beneficial for health (indicating adequate knowledge), most of them (54.5%) still consumed regular pasta [15]. In Australia, Foster et al. (2020) found that only 8% of the study population met dietary recommendations for whole grain intake, despite 75% being aware of their health benefits [16]. In line with previous studies, although the majority of participants were aware of the health benefits of whole‑grain bread reflected by correct responses to questions assessing the health differences between whole‐grain and white bread (Q3, 73.0% correct) and the protective role of whole‐grain bread against chronic diseases (Q4, 60.1% correct), their actual consumption remained low. This finding may be explained by participants' limited ability to distinguish whole‑grain bread from white bread as reflected by incorrect responses to questions 1 and 2 (62% and 63%, respectively) as well as the presence of perceived barriers. This finding clearly highlights the gap between knowledge and practice regarding whole‐grain bread consumption. Notably, among the 340 students who correctly acknowledged the health benefits of whole‑grain bread, 69.4% still consumed it rarely or only 1–2 times per month, quantitatively confirming this knowledge–action gap.

### 4.2 Perceived Barriers to Whole‐Grain Bread Intake

Limited accessibility (68.3%), hard texture (56.4%), higher price (54.4%), and unfavorable taste (51.1%) were the most frequently reported barriers (see Results for full list). Students who reported rare consumption of whole‑grain bread had significantly higher barrier scores than those consuming it ≥ 1–3 times per week (Table 2). Comparable findings were observed in previous studies emphasizing the multifactorial nature of perceived barriers, including sensory, economic, and accessibility‐related factors. Price, taste, sustainability, and versatility were reported as the main barriers to whole grain consumption in an Italian population studied by Zanini et al [31]. They also found positive correlations between the ‘taste' and ‘versatility' barriers and higher consumption frequency [31]. The perceived barriers of whole‐grain bread consumption in an Iranian university students, were included classmates' preferences (96.2%), family dietary patterns (91.8%), lack of appeal (72.8%), limited accessibility (70.5%), and high prices (43.9%) [13]. In contrast, Wongprawmas et al., studying Cornell University students in the U.S., reported that price and availability were not barriers to whole‐grain pasta consumption, likely because, at Cornell dining venues, whole‐grain pasta is typically offered at the same price as regular pasta through the Eating Well with Cornell Dining program [15]. A qualitative study on Iranian adults found that although the majority of participants (70%) believed whole traditional bread was healthier and more delicious, low availability, darker color, higher price, and lifestyle changes prevented them from replacing white‐bread [39]. Foster et al. (2020) in Australia reported that education level, cost, taste, and availability were the main barriers to whole grain consumption. Overall, their findings indicate that while participants' general nutritional knowledge was moderate to high, perceived barriers were common and varied, which could be related to differences in dietary behaviors and food choices [16].

### 4.3 Associations With Broader Dietary Habits

Prior studies indicate that refined‑bread consumers are more likely to skip meals and prefer fast foods [13], health‐conscious individuals more often choose whole‐grain pasta [15], and regular breakfast consumers have higher whole‐grain intake [40].

Consistent with previous studies, participants who reported consuming whole‑grain bread tended to have higher daily meal intake and lower consumption of sugar‐sweetened beverages (Table 2). The frequency of whole‑grain bread consumption showed weak but statistically significant positive correlations with knowledge score and intake of fruits, vegetables, pulses, dairy, eggs, white meat, and red meat, and weak negative correlations with barriers score and consumption of sweet/salty snacks (correlation coefficients < 0.5) (Table 2, Table 4). Additionally, a higher knowledge score was associated with slightly higher odds of dairy intake (OR = 1.194), while higher barrier scores were associated with lower odds of vegetable (OR = 0.943), fruit (OR = 0.941), and dairy intake (OR = 0.938), but with higher odds of sweet/salty snack consumption (OR = 1.067) (Table 6). Overall, these findings indicate modest associations between knowledge, perceived barriers, and dietary behaviors, and suggest that students with healthier dietary profiles tended to include whole‑grain bread more frequently in their routine diet.

### 4.4 Non‐Significant Demographic and Lifestyle Factors

A statistically significant but trivial positive correlation was observed between the number of main meals per day and whole‑grain bread consumption (r = 0.124, *p* = 0.003; Table 2). This trivial correlation probably mirrors the clustering of health‑protective habits, given that a higher number of main meals is normally linked to better overall diet quality rather than functioning as an independent driver of whole‑grain intake [41, 42]; simultaneously, a larger meal frequency may amplify total fiber intake marginally [43], which could partly underlie the weak positive signal witnessed in this study.

No significant gender differences in consumption, knowledge, or barriers were found (Tables 2 and 3), in contrast with evidence linking higher whole‐grain intake to female gender [44, 45], suggesting that product‐specific barriers affect both sexes equally in this medical‐university setting.

Age and BMI were also unrelated to consumption. The narrow age range of a student sample likely underlies the absent age gradient, whereas older populations exhibit increased whole‐grain intake with age [46]. The lack of BMI association aligns with some cross‐sectional studies reporting weak or null links [47, 48], though longitudinal data suggest an inverse relationship between whole‐grain consumption and weight gain [48, 49].

Daytime and nighttime sleep showed only trivial correlations (daytime: r = −0.10, *p* = 0.02; nighttime: r = 0.088, *p* = 0.04). These negligible associations are consistent with prior reports in young adults [50], while some evidence indicates that poor sleep quality, rather than duration alone, may adversely affect overall dietary patterns [51, 52].

Type of regular cooking oil, history of chronic diseases, and number of daily snacks were not significantly associated with whole‐grain bread intake. Cooking oil choice is a separate dietary behavior shaped primarily by cultural and economic factors, and no prior study has identified a direct link [53]. The absence of association with disease history is expected given the low prevalence of chronic conditions in this young cohort [54, 55]. For snacks, this null association likely reflects the predominance of refined, energy‑dense items in this cohort, consistent with the view that snacking habits are often distinct from staple grain choices, despite some evidence linking frequent snacking to higher whole‑grain intakes in general adult populations [56]. Collectively, these findings indicate that whole‑grain bread intake in this population is driven predominantly by nutritional knowledge and perceived barriers rather than by a broader health‑conscious lifestyle profile.

### 4.5 Public Health Implications and Future Directions

**Figure 1:**
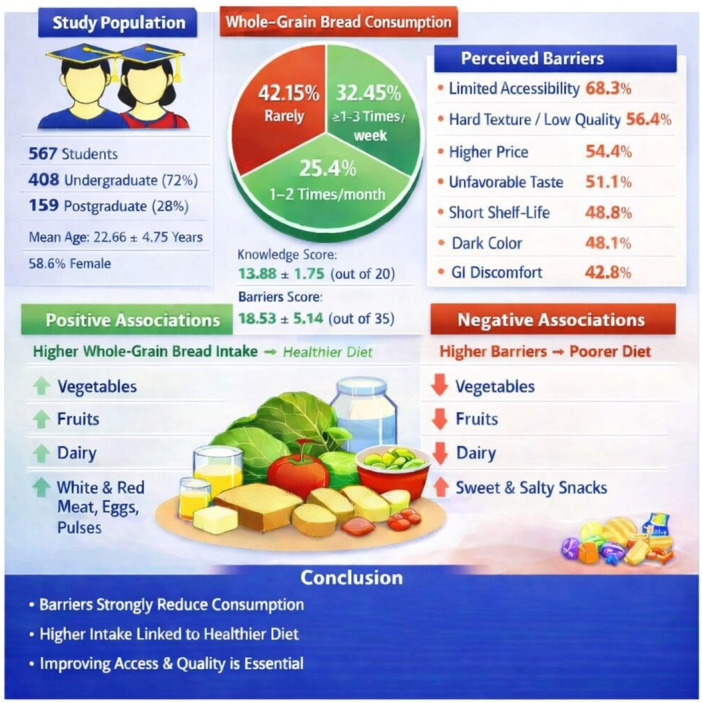
Graphical summary of whole‐grain bread consumption frequency, mean knowledge and barrier scores, most commonly reported barriers, and the direction of associations with dietary habits.

Public health strategies should therefore extend beyond consumer education to include structural interventions, such as improved cafeteria access, pricing policies, and product reformulation, given that whole‐grain breads, despite comparable sodium content, offer substantially higher fiber and micronutrient levels [57]. The key findings of this study are visually summarized in the graphical abstract (Figure 1).

### 4.6 Strengths and Limitations

This study has limitations. Data came from a single medical sciences university, so generalizability to non‐health students is limited and baseline nutrition knowledge may be higher than in other cohorts. Whole‐grain bread intake was measured as frequency, not quantity. Anthropometric data were self‐reported, which may introduce bias in BMI estimates. The cross‐sectional design prevents causal inference. Nonetheless, strengths include the validated, face‐to‐face questionnaire (CVI/CVR = 1.0), the simultaneous assessment of knowledge and barriers, and the novel integration of bread consumption with broader dietary habits.

## 5 Conclusion

Despite moderate‑to‑high awareness of the health benefits of whole‑grain bread, actual consumption among university students remained low, highlighting a knowledge–practice gap linked to perceived barriers such as accessibility, texture, and price. Whole‑grain bread intake was positively associated with healthier dietary habits, suggesting that its promotion may be one component of broader strategies to support healthy eating in this population. Because whole‑grain breads offer substantially more fiber and micronutrients than white bread, even with comparable sodium content, relevant policy measures should extend beyond consumer education. Future studies should employ quantitative dietary assessments and include more diverse populations to enhance generalizability and explore potential causal relationships.

## Author Contributions

**Ali Keshavarz:** conceptualization, investigation, data curation, writing – review and editing, writing – original draft, methodology. **Fatemeh Borazjani:** conceptualization, writing – original draft, writing – review and editing, supervision, methodology. **Faezeh Moeini Badi:** writing – original draft. **Kambiz Ahmadi Angali:** formal analysis, writing – review and editing. **Mohammad Parsa Bayat:** data curation, investigation, writing – review and editing. **Samaneh Hajjarzadeh:** supervision, writing – original draft, conceptualization, writing – review and editing.

## Ethics Statement

The study was approved by the Ethics Committee of Jundishapur University of Medical Sciences in accordance with the Declaration of Helsinki (IR.AJUMS.REC.1404.137). Written informed consent was obtained from all participants prior to enrollment.

## Conflicts of Interest

The authors declare no conflicts of interest.

## Data Availability

The data that support the findings of this study are available from the corresponding author upon reasonable request.
